# Sorsby fundus dystrophy (SFD): A narrative review

**DOI:** 10.1097/MD.0000000000030595

**Published:** 2022-09-23

**Authors:** Georgios Tsokolas

**Affiliations:** a Medical Retina and Uveitis Fellow, Moorfields Eye Hospital, London, United Kingdom

**Keywords:** Bruch membrane, disease modeling, Drusen, hereditary retinal dystrophy, macula

## Abstract

Sorsby fundus dystrophy (SFD) is a rare autosomal dominant disorder with complete penetrance affecting the macula. This is caused by a mutation in the TIMP-3. This objective narrative review aims to provide an overview of the pathophysiology, current treatment modalities, and future perspectives. A literature search was performed using “PubMed,” “Web of Science,” “Scopus,” “ScienceDirect,” “Google Scholar,” “medRxiv,” and “bioRxiv.” The molecular mechanisms underlying SFD are not completely understood. Novel advancements in cell culture techniques, including induced pluripotent stem cells, may enable more reliable modeling of SFD. These cell culture techniques aim to shed more light on the pathophysiology of SFD, and hopefully, this may lead to the future development of treatment strategies for SFD. Currently, no gene therapy is available. The main treatment is the use of anti-vascular endothelial growth factors (anti-VEGF) to treat secondary choroidal neovascular membrane (CNV), which is a major complication observed in this condition. If CNV is detected and treated promptly, patients with SFD have a good chance of maintaining a functional central vision. Other treatment modalities have been tried but have shown limited benefit, and therefore, have not managed to be more widely accepted. In summary, although there is no definitive cure yet, the use of anti-VEGF treatment for secondary CNV has provided the opportunity to maintain functional vision in individuals with SFD, provided CNV is detected and treated early.

## 1. Introduction

Sorsby fundus dystrophy (SFD) is a rare hereditary macular dystrophy with an autosomal dominant mode of inheritance and full penetrance. It was first described in the literature published in 1949 by Sorsby et al^[1]^ and named after the leading author of this manuscript.^[1]^ Patients with this condition may become symptomatic during their second decade of life, however, the average onset of the disease is usually during the fourth to fifth decade of life.^[1,2]^ Prior to the era of anti-vascular endothelial growth (anti-VEGF) injections, SFD led to severe bilateral visual reduction and blindness.^[1,2]^

The aim of this narrative review is to provide a descriptive overview of this inherited genetic disorder.

This descriptive review also aims to answer the following questions:

A. What is the prevalence of SFD?
B. What are the underlying pathophysiological mechanisms of SFD?
C. What are the signs and symptoms of the disease?
D. What are the current treatment modalities available for SFD and how effective are they?
E. What potential future strategies can be developed to treat the disease?

## 2. Materials and Methods

A literature search was performed using “PubMed,” “Web of Science,” “Scopus,” “ScienceDirect,” “Google Scholar,” “medRxiv,” and “bioRxiv.” The main keywords for the literature search were “Sorsby Fundus Dystrophy,” “Choroidal Neovascular Membrane (CNV),” “Photodynamic Therapy (PDT),” “Tissue Inhibitor of Metallproteinase-3 (TIMP-3),” “Vascular Endothelial Growth Factor (VEGF),” “Bevacizumab,” “Ranibizumab,” “Triamcinolone,” “ARPE-19 cells,” and “induced pluripotent stem cells (iPSC).” The author attempted to collect data from manuscripts that were mainly published in the past 15 years so that the most recent and up-to-date information on SFD could be incorporated into this manuscript. Nevertheless, published manuscripts >15 years of age were also used, as they contained pertinent information about SFD. The literature search yielded approximately 300 manuscripts. To comply with the maximum number of references, 101 articles were selected.

As this is a narrative review, no ethical approval was required.

## 3. Narrative review findings

### 3.1. Epidemiology

Due to the paucity of SFD cases, no large epidemiological studies exist that calculate the prevalence of the disease in the general population. The only manuscript that attempts to calculate the prevalence is the manuscript by Christensen et al published in 2017.^[3]^ The authors of this study estimated the prevalence of SFD to be 1 in 220,000.^[3]^ Since the condition is inherited in an autosomal dominant manner with complete penetrance, there is no gender predilection. Hence, both men and women can be equally affected by this rare hereditary macular dystrophy.

### 3.2. Signs and symptoms

Patients with this condition may become symptomatic during their second decade of life; however, the average onset of the disease is usually during the fourth to fifth decade of life.^[1,2]^ Deposition of drusen within the posterior pole is typically observed during the initial stages of the disease.^[4,5]^ Reticular drusen and pseudodrusen may also be observed on dilated fundoscopy.^[2]^ In more advanced stages of the disease, formation of a secondary choroidal neovascular membrane (CNV) or geographic atrophy can be observed.^[4,5]^ Because the primary affected location of the retina is the macula, the main symptoms include distortion, reduced distant and near vision, impaired color vision, and nyctalopia.^[2,4–6]^ The peripheral retina may be involved during the process of the disease and loss of ambulatory vision may ensue during the 7th decade of life.^[2,4–6]^ Prior to the era of anti-VEGF injections, SFD led to severe bilateral visual reduction and blindness.^[1,2]^

### 3.3. The role of TIMP-3 gene—a brief overview

SFD is attributed to mutations affecting TIMP-3.^[3,4]^ This gene is 1 of the 4 genes encoding enzymes that serve as inhibitors of the matrix metalloproteinases (MMP).^[3,7]^ The cluster of these 4 genes demonstrates a robust tertiary molecular structure induced by the presence of 6 intramolecular disulfide bonds forming between 12 cysteine residues.^[3]^ All 4 genes halt the breakdown of the extracellular matrix due to their N-terminal domain.^[3,8–10]^ This is of paramount importance for a wide range of physiological processes at a cellular level, including wound healing, angiogenesis, and tissue remodeling.^[3]^ In addition, TIMP-3 seems to be able to inhibit the interaction of VEGF with its receptor VEGF receptor 2 (VEGFR2) by directly binding to the receptor, thus preventing angiogenesis.^[3,11]^ Nevertheless, it is still unclear whether TIMP-3 gene is involved in the apoptosis of retinal pigment epithelial (RPE) cells and how this can affect SFD.^[3]^ Unlike TIMP-1, TIMP-2, and TIMP-4, which are soluble, TIMP-3 is insoluble.^[3,12,13]^

### 3.4. Mutations in TIMP-3 gene

Based on the author’s literature review and on the manuscript by Christensen et al,^[3]^ 18 mutations of the TIMP-3 gene have been implicated in the manifestation of SFD.^[3,6,14–29]^ In the United Kingdom, most patients with SFD carry the Ser204Cys mutation in exon 5 of the TIMP-3 gene.^[5]^

The presence of different variants in TIMP-3 makes SFD a condition that exhibits heterogeneity in the severity of different clinical phenotypes.^[3]^ Although most mutations are located closer to the C-terminus of the protein, there are mutations affecting the N-terminus as well, and the severity of the disease cannot be easily correlated with the affected amino acid.^[3,14–16,21,22,24,29]^ Some authors suggest that some additional risk factors may affect the severity of SFD, such as smoking or age-related macular degeneration (AMD) genetic factors.^[3,17,30]^

Most mutations described in the manuscripts referenced above affecting the amino acid cysteine leading to the dimerization of TIMP-3 variants observed with these mutations.^[3,15,18,25,27,28]^ However, mutations not involving the amino acid cysteine have also been described.^[3,17,23]^ It is also unclear whether TIMP-3 variants retain their ability to inhibit MMP, although most studies advocate this.^[3,11,18,31]^ It is postulated that loss of the ability to inhibit MMP leads to a more severe phenotype, but some studies dispute this hypothesis.^[3,17,32]^

Another controversial topic is whether various TIMP-3 variants preserve the ability to impede the interaction between VEGF and its receptor.^[3,33,34]^ As mentioned above, TIMP-3 seems to be able to inhibit the interaction of VEGF with its receptor VEGF receptor 2 (VEGFR2) by directly binding to the receptor, thus preventing angiogenesis.^[3,11]^ It is postulated that this may represent a pivotal step leading to formation of CNV.^[3]^ Nevertheless, patients with SFD do not seem to have an abnormal choroidal structure, as other studies conducted using mouse animal models have shown.^[3,35]^ Hence, SFD variants may still retain the ability to impede the VEGF signaling cascade to some extent.^[3,35]^

Another interesting point is the observations of the manuscript by Meunier et al.^[36]^ The authors described a series of SFD patients from 2 unrelated families, where not only the eyes were affected, but the patients also exhibited pulmonary involvement.^[36]^ Of note, pulmonary involvement has been described in mouse models with TIMP-3 mutations.^[37]^ Meunier et al also posed the question of whether the substitution of a smaller amino acid with a larger amino acid may lead to a more significant impact in the TIMP-3 protein structure, leading to a more severe clinical phenotype of SFD.^[3,36]^ However, the early onset of disease associated with the Ser179Cys variant (where again a smaller amino acid is replaced by a larger amino acid) suggests that age of onset is not solely determined by the change in the size of the variant amino acid.^[3]^ Therefore, the hypothesis proposed by Meunier et al could not be confirmed.^[3,36]^

In addition, another study using mouse models proposed that mutations in TIMP-3 can potentially lead to osteoporosis.^[38]^ Tsokolas et al^[5]^ described 2 sisters who suffered from SFD that were referred for lung function tests and bone densitometry, which were both normal. Hence, it could be postulated that SFD can be perceived as a syndromic disease with potentially affected extraocular tissues.

In summary, there are still unanswered questions regarding TIMP-3 expression. Solving these mysteries around TIMP-3 can open new pathways in the development of therapeutic strategies for SFD.^[3]^

### 3.5. Pathophysiology associated with TIMP-3 mutations

TIMP-3 is expressed primarily in the RPE and choroidal endothelial cells.^[3,39–41]^ It is also an integral part of Bruch membrane.^[3,42]^

Studies have suggested that increasing age may lead to increased deposition of TIMP-3 on the Bruch membrane.^[3,43]^ Other studies have suggested that this is because of increased deposition of TIMP-3 molecules rather than true overexpression of the gene.^[3,44,45]^ This is supported by using immuno-staining.^[3,40]^ Despite the fact that TIMP-3 gene is expressed in both RPE and choroidal endothelial cells, the previously mentioned studies postulate that the accumulation of TIMP-3 protein originates most likely from the RPE rather than the choroidal endothelium, making the RPE the primary location of the changes that eventually lead to SFD.^[3]^

Mutations in TIMP-3 result in the accumulation in the drusen-type of deposits at the level of Bruch membrane observed clinically on fundoscopy in SFD patients.^[3,18,32]^ The subsequent thickening of Bruch membrane eventually affects RPE function. The subsequent disruption of the RPE–retina barrier may result in macular atrophy or CNV formation.^[3,18,32]^

At this stage, it is imperative to highlight that TIMP-3 protein accumulation is not only observed in SFD but also in other pathological conditions, including retinitis pigmentosa and AMD, where there is abnormal material deposition at the level of Bruch membrane.^[3,40,43,46,47]^ Therefore, understanding the underlying pathophysiological mechanisms associated with TIMP-3 variants will shed more light into gaining more insight about SFD and other TIMP-3 associated retinopathies as well and will help devise treatment strategies in the future.^[3]^

### 3.6. Cell culture models to understand SFD

Culture of RPE cells has been used for decades to better understand their roles and functions.^[3]^ In the past, different types of tissues were used for this purpose.^[48–52]^ In recent years, RPE cells are cultured on porous materials.^[3,53]^

There are numerous advantages to the use of porous materials for RPE cell cultures. One such advantage is that the cultured cells exhibit polarization of their membranes, which allows the study and quantification of different secreted proteins and membrane receptors.^[3,54–59]^ In addition, the use of porous material allows the study of the interactions between the RPE and choroid, hence the study of the blood–retinal barrier.^[3,60,61]^ Finally, RPE cells interact with the photoreceptors and play a pivotal role in the regeneration of the photoreceptor outer segment during the visual cycle. This interaction can also be using porous materials.^[3,62]^ These advantages have made RPE cell cultures very useful in modeling conditions, in which there is a breakdown in the normal RPE function and its interactions with the retina, including drusen formation.^[3,63,64]^ Such ex-vivo studies have provided important advancements in culturing RPE cells and have highlighted the necessity to use RPE cells derived from live patient human tissue that can provide more reliable insight into different aspects of RPE pathophysiology.^[3]^

A very important novel cell culture technique that seems to be quite promising for studying RPE cells is the use of pluripotent stem cells that can differentiate into RPE cells.^[3]^ Pluripotent stem cells preserve the ability to differentiate in any type of cell originating from the 3 germ layers.^[3]^ Pluripotent stem cells were first isolated in 1981, and they derived from mouse embryonic stem cells.^[3,65,66]^ Human embryonic stem cells followed 17 years later.^[3,65,66]^ Ever since, studies have achieved to generate RPE cells from human embryonic stem cells via direct and spontaneous differentiation.^[3,67–70]^

RPE cells originating from pluripotent stem cells exhibit morphological features that are identical to those of the typical human RPE cells.^[3,68,70]^ Furthermore, studies suggest that these cells are superior to other cell lines for modeling human RPE cells.^[3,70–73]^ These attributes have allowed the use of pluripotent stem cell derived RPE cells in the formation of study models of macular dystrophies and in human clinical trials for AMD and Stargardt disease.^[3,70,74]^

Another ground-breaking evolution is the ability to generate pluripotent stem cells from somatic cells, known as iPSCs.^[3]^ This technique allows the propagation of embryonic stem cells with the same characteristics shared by conventional pluripotent cells.^[3,75]^ Studies have demonstrated the ability to produce RPE cells from iPSCs.^[3,76–80]^ RPE cells originating from iPSCs seem to be superior to those originating from embryonic stem cells, because they carry the same genetic information in situ (including mutations) with the patient’s somatic cells (e.g., skin fibroblasts) from which they are produced.^[3,77]^

The above attributes demonstrated by iPSCs have been used to model AMD and other retinal dystrophies, including Best disease and retinitis pigmentosa.^[3,77,81,82]^ Nevertheless, iPSCs have not been used to model SFD or study various TIMP-3 mutations.^[3]^ In addition, to date, non-RPE cells or non-human cells have been used in various studies to emulate SFD, but these cell types are not reliable for studying the underlying pathophysiological mechanisms associated with SFD and TIMP-3 mutations.^[3,11,17,18,31,32,83–85]^ Therefore, exploiting the novel advancements in cell culture techniques can open new pathways for formulating patient-derived RPE cell types that can allow a more reliable and realistic depiction of the various TIMP-3 variants under in vitro conditions.^[3]^ Studies focusing on the TIMP-3 gene and SFD should incorporate the evaluation of TIMP-3 expression, assessment of the role of the RPE in maintaining the RPE–retina barrier, their role in the renewal of the photoreceptor outer segment during the visual cycle and their interaction with the choroidal vascular network.^[3]^ Such studies will allow us to elucidate the molecular mechanisms that may result in CNV formation or geographic atrophy which are the hallmark clinical features of SFD.^[3]^

### 3.7. Treatment of SFD

To date, there is no definitive treatment for TIMP-3 mutations leading to SFD. Prior to the introduction of anti-VEGF agents in clinical practice, CNV formation used to lead to rapid irreversible loss of central vision and legal blindness.^[2]^ Prior to anti-VEGF treatment, vitamin A was administered to treat SFD related nyctalopia.^[2,86]^ Low doses of Vitamin A were not efficient in the treatment of nyctalopia, whereas higher doses induced liver toxicity.^[2,86]^ Hence, Vitamin A has not been widely adopted for the treatment of night blindness associated with SFD.

CNV formation is the leading cause of central vision loss in patients with SFD. Prior to the introduction of anti-VEGF agents in clinical practice, thermal photocoagulation has been used as an attempt to treat CNV.^[2,6,87]^ However, thermal photocoagulation did not improve visual acuity and aggressive recurrence of CNV was observed as well.^[2,6,87]^ Therefore, thermal photocoagulation has been deemed obsolete as treatment modality for was PDT verteporfin.^[2]^ PDT was used as a stand-alone therapy or combined with intravitreal corticosteroid injection.^[2]^ Nevertheless, this treatment approach showed very limited benefits, was unpredictable and was eventaully abandoned.^[2,6,19,88,89]^

Five years later, anti-VEGF treatment was introduced in clinical practice, which this revolutionized the treatment of CNV secondary to SFD. There have been quite a few publications that have evaluated the efficacy and safety of anti-VEGF agents in treating CNV secondary to SFD and they have been summarized in a comprehensive systematic review by Baston et al published in 2021.^[2,5,6,19,24,90–99]^

A systematic review by Baston et al^[2]^ described a mean follow-up period of 54 months for patients treated for SFD. If CNV is recognized and treated early, this systematic review demonstrated that 51% of eyes can preserve an adequate reading and driving visual acuity (measured as Snellen decimal visual acuity) of at least 0.5.^[2]^ Furthermore, prompt treatment allowed 67% of eyes to maintain a functional level of vision using reading aids (Snellen decimal visual acuity of at least 0.2).^[2,5,6,19,24,90–96]^ If, however, an established disciform scar has ensued, the use of anti-VEGF treatment is of limited benefit.^[2]^ It must also be pointed out that even after prompt treatment, SFD can still progress either due to geographic atrophy or due to macular scar formation.^[2,98]^ In fact, Kaye et al described a linear decrease in VA of approximately 0.1 Logarithm of Minimum Angle of Resolution units per year until scar formation after a 5-year follow-up period.^[2,98]^

All the above published manuscripts have significant limitations, namely their retrospective nature and the limited number of patients/eyes treated.^[2,5,6,19,24,90–99]^ Some of them have also small follow-up periods,^[90,92,96,97]^ whereas others have longer follow-up periods.^[2,5,6,98,99]^ In addition, there is no consensus on the optimum pattern of anti-VEGF injections, that is, whether a treat and extend (T&E) protocol would be preferable to a pro-re-nata (PRN) approach. Kaye et al and Tsokolas et al advocated a T&E approach, whereas Baston et al did not.^[2,5,98]^ Ideally, prospective studies with larger number of patients that will be divided into the 2 above treatment groups should be conducted. Nevertheless, such an endeavor is quite challenging to come into fruition due to the rarity of SFD patients, which will subsequently raise doubts about the cost-effectiveness of such an endeavor. Despite the lack of consensus in the pattern of injections, all manuscripts highlight the importance of prompt recognition and treatment of CNV secondary to SFD and acknowledge that the introduction of anti-VEGF agents was a game changer in the clinical outcome of CNV secondary to SFD.^[2,5,6,19,24,90–99]^

Recently, Spaide published a case report of an SFD patient who was followed-up for approximately 16 years and treated with a series of intravitreal injections of triamcinolone.^[100]^ The patient originally presented in 2003 with visual acuities of 20/25 and 20/400 right and left eye respectively.^[100]^ PDT was originally tried with no benefit, which confirmed the observations of other published manuscripts.^[2,6,19,88,89,100]^ The left eye had no further treatment due to permanent foveal structural damage, whereas the right eye was further treated with series of intravitreal injections of triamcinolone in 3 to 4 monthly intervals and retained an excellent acuity.^[100]^ The author postulates that the anti-inflammatory effect of triamcinolone may be able to modify the secretion of VEGF and tumor necrosis factor-α (TNF-α) regulated by TIMP-3 gene.^[100]^ This case report has a long follow-up period, but it has 2 main weaknesses: its retrospective nature and the fact that only 1 eye was treated. Hence, this case report does not provide robust data to advocate the use of intravitreal triamcinolone as a mainstream treatment for CNV secondary to SFD. In addition, intravitreal steroids such as triamcinolone increase the risk of cataract formation and steroid response/glaucoma. These 2 additional risk factors must be considered as they may pose an additional visual burden in patients with SFD, who are already at risk of losing central vision in a relatively young age. Finally, as with anti-VEGF treatment, there is no benefit with steroid treatment when a disciform scar has already formed, as with the patient’s left eye. Hence, larger prospective studies are needed to provide substantial evidence that could make the use of triamcinolone mainstream. However, such an endeavor is quite challenging to come into fruition due to the rarity of patients with SFD, which will subsequently raise doubts about the cost-effectiveness of such an endeavor.

Spaide also described the use of adalimumab in the same patient described above for the treatment of SFD.^[101]^ After a series of triamcinolone intravitreal injections, the treatment was switched to bevacizumab, but CNV became active.^[101]^ Then, intravitreal triamcinolone was introduced again, and the patient responded with minimal signs of exudation and hemorrhage.^[101]^ Due to long-term complications associated with intravitreal triamcinolone, the treatment was switched to subcutaneous adalimumab.^[101]^ The total follow-up period after the initiation of adalimumab was 18 months. Throughout this period, CNV in the right eye remained inactive and the final visual acuity in the right eye was 20/20.^[101]^ Since TIMP-3 can regulate the secretion of TNF-α locally, the author suggests that the use of anti-TNF-α agents such as adalimumab may offer a molecularly oriented approach to treatment and warrants further evaluation.^[101]^ Therefore, larger prospective studies are required to elucidate whether adalimumab could be a good alternative treatment for CNV secondary to SFD. However, as mentioned previously, such prospective studies require large numbers of patients and prolonged follow-ups, which is very difficult to achieve given the paucity in numbers of patients with SFD. In addition, biologic agents such as adalimumab have side effects, mainly immunosuppression. In addition, application for special funding is usually required for biologics, and this process can be quite time consuming; hence, patients with SFD will still need prompt treatment with anti-VEGF agents in the interim, until adalimumab is introduced.

In summary, anti-VEGF agents have changed the visual outcomes of CNV secondary to SFD. They remain the cornerstone of treatment of patients with SFD that develop CNV. Intravitreal steroids and biologics have also been reported, but the currently available data are limited to support their wider use in the treatment of CNV associated with SFD.

## 4. Conclusions and final comments

SFD is a rare inherited retinal dystrophy for which no definitive treatment is currently available. There are still many unanswered questions regarding the underlying molecular pathophysiological mechanisms associated with TIMP-3 mutations. Current cell types and animal models used to understand these mechanisms are not fully reliable. With novel advancements in cell culture techniques and the emergence of iPSCs, there is hope that SFD can be modeled more accurately and reliably. This may open new pathways for developing treatment strategies for SFD which can target TIMP-3.

Currently, the main aim is to treat complications of SFD, mainly CNV formation. Prior to the introduction of anti-VEGF agents into clinical practice, CNV was untreatable, leading to irreversible foveal damage and subsequent central visual loss. Anti-VEGF treatment has allowed for the preservation of functional vision of individuals with SFD that develop CNV. Patients with SFD must be warned about this significant complication associated with their diagnosis and they must be educated in the regular use of the Amsler grid so that they can detect metamorphopsia promptly and seek immediate help in their local eye casualty service. This will increase the chances of prompt treatment with anti-VEGF agents and subsequent preservation of vision and quality of life. Patients with SFD should also be educated to avoid smoking and follow a diet rich in fruit and vegetables, as these 2 lifestyle habits can slow down the retinal degeneration induced by TIMP-3 mutations.

Intravitreal corticosteroids and subcutaneous adalimumab were recently described in 2 case reports as treatment modalities for CNV secondary to SFD. However, supporting data are limited to advocate their wider use, and their efficacy merits further study.

Therefore, anti-VEGF agents remain the cornerstone of treatment for CNV secondary to SFD, provided that this complication is recognized and attended to early.

## Author contributions

**Conceptualization:** Georgios Tsokolas.

**Methodology:** Georgios Tsokolas.

**Writing – original draft:** Georgios Tsokolas.

**Writing – review & editing:** Georgios Tsokolas.
